# MoccaDB - an integrative database for functional, comparative and diversity studies in the *Rubiaceae *family

**DOI:** 10.1186/1471-2229-9-123

**Published:** 2009-09-29

**Authors:** Olga Plechakova, Christine Tranchant-Dubreuil, Fabrice Benedet, Marie Couderc, Alexandra Tinaut, Véronique Viader, Petra De Block, Perla Hamon, Claudine Campa, Alexandre de Kochko, Serge Hamon, Valérie Poncet

**Affiliations:** 1 UMR DIAPC, IRD, 911 avenue Agropolis, BP 64501, 34394 Montpellier Cedex 5, France; 2 CIRAD TA C 37/D, Campus International de Baillarguet 34398 Montpellier Cedex 5, France; 3 UMR DIAPC, INRA, Domaine de MelgueiI, Chemin de Mézouls, 34130 Mauguio, France; 4 National Botanic Garden of Belgium, Domein van Bouchout, 1860 Meise, Belgium

## Abstract

**Background:**

In the past few years, functional genomics information has been rapidly accumulating on Rubiaceae species and especially on those belonging to the *Coffea *genus (coffee trees). An increasing number of expressed sequence tag (EST) data and EST- or genomic-derived microsatellite markers have been generated, together with Conserved Ortholog Set (COS) markers. This considerably facilitates comparative genomics or map-based genetic studies through the common use of orthologous loci across different species. Similar genomic information is available for e.g. tomato or potato, members of the Solanaceae family. Since both Rubiaceae and Solanaceae belong to the Euasterids I (lamiids) integration of information on genetic markers would be possible and lead to more efficient analyses and discovery of key loci involved in important traits such as fruit development, quality, and maturation, or adaptation. Our goal was to develop a comprehensive web data source for integrated information on validated orthologous markers in Rubiaceae.

**Description:**

MoccaDB is an online MySQL-PHP driven relational database that houses annotated and/or mapped microsatellite markers in Rubiaceae. In its current release, the database stores 638 markers that have been defined on 259 ESTs and 379 genomic sequences. Marker information was retrieved from 11 published works, and completed with original data on 132 microsatellite markers validated in our laboratory. DNA sequences were derived from three *Coffea *species/hybrids. Microsatellite markers were checked for similarity, *in vitro *tested for cross-amplification and diversity/polymorphism status in up to 38 Rubiaceae species belonging to the Cinchonoideae and Rubioideae subfamilies. Functional annotation was provided and some markers associated with described metabolic pathways were also integrated. Users can search the database for marker, sequence, map or diversity information through multi-option query forms. The retrieved data can be browsed and downloaded, along with protocols used, using a standard web browser. MoccaDB also integrates bioinformatics tools (CMap viewer and local BLAST) and hyperlinks to related external data sources (NCBI GenBank and PubMed, SOL Genomic Network database).

**Conclusion:**

We believe that MoccaDB will be extremely useful for all researchers working in the areas of comparative and functional genomics and molecular evolution, in general, and population analysis and association mapping of Rubiaceae and Solanaceae species, in particular.

## Background

Accumulation of available genetic markers directly contributes to advances in marker-assisted genetic studies with a wide range of applications such as detection and identification of individual genes and/or quantitative trait loci (QTL), or exploration of the genetic diversity and population structure with regard to natural variations [1-3]. The recent and rapid accumulation of sequence resources, mainly from crop species, ensures an improvement of the genetics approach in combination with the comparative genomics. The extension of these genome resources to their close relatives as well as to more distant genera greatly facilitates the elucidation of evolutionary histories. This elucidation involves the discovery and study of key orthologous loci, phylogeny reconstruction and a variety of other biological questions.

The Rubiaceae family is the fourth largest family of flowering plants but, except for rare species such as *Kadua centranthoides *Hook. & Arn. [as *Hedyotis centranthoides*]*and Kadua affinis *Cham. & Schltdl. [as *Hedyotis terminalis*] (Levesque MP, Twigg RW, Motley T, Katari MS, Dedhia NN, O'Shaughnessy AL, Balija V, Martienssen RA, McCombie RW, Benfey P et al: Expressed tag sequences from *Hedyotis centranthoides *and *Hedyotis terminalis *flowers - Stage 2 (NYBG), accessions available from  2003), most of the genomic information has been generated from the major economic crop species of the *Coffea *genus, cultivated throughout the tropics: *C. arabica *L. and *C. canephora *Pierre ex A.Froehner, the Arabica and Robusta coffee trees, respectively. They are thus used as molecular models for the Rubiaceae. Integrative information of genomic and genetic knowledge acquired for these plants can be further extended to other *Coffea *species but also to other economically important Rubiaceae genera used in medicine (e.g. *Cinchona*, which produces quinine, is used as a cure for malaria), and in horticulture (e.g. many genera, including *Gardenia*, *Ixora*, *Pentas*, *Mussaenda *and *Sherardia*, are well known ornamentals [4]).

Among PCR-amplified markers, microsatellite (or simple sequence repeat, SSR) markers are commonly used in large-scale genomic studies owing to their ubiquitous distribution in both protein-coding and non-coding regions and the high degree of length polymorphism among individuals [5]. The *C. canephora *microsatellites were screened in a leaf and fruit EST database [2] and in a *C. canephora *BAC sequence [6]. The overall SSR density has been estimated as one SSR every 7.73 kb and one SSR every 4.1 kb, in the ESTs and in the genomic sequences, respectively [2,6]. However, although microsatellites are distributed ubiquitously throughout the *Coffea *genome, only a few of them are suitable for designing informative markers with properties such as strong and specific amplified fragment after PCR and easy scoring of allele sizes, high heterozygosity and/or known position along a linkage map.

Functional genomics is particularly promising for identifying genes involved in a variety of biological functions, which include pathways related to the coffee beverage quality such as synthesis of caffeine, sugars, lipids and chlorogenic acids, but also those related to fruit development. The use of markers directly targeting expressed genes important for each specific trait would be beneficial to these studies. Due to the ongoing sequencing of expressed genes from different plant organs, it is now possible to develop EST-SSR markers for important traits, like fruit properties.

Previous publications [1,2,7,8] and the present study have revealed that coffee EST-SSR and SSR markers show a high level of transferability across distantly related species, thereby providing additional markers for orphan Rubiaceae species.

Although the genomic data available on coffee plants are rapidly increasing, they are often isolated and scattered and rarely available online. In the present study, an effort has been made to create a centralized access to both published and original new data on evolutionarily conserved and validated markers. Integrated comprehensive information system and bioinformatics tools are provided, which will be useful for the research community working on plant genetics and evolution of coffee tree related organisms.

## Construction and content

### Data source

The data retrieval and compilation for MoccaDB has involved the following steps: (1) extraction of data from various sources (publications, public databases etc.); (2) development and testing of additional new markers in Rubiaceae species; (3) compilation, elimination of marker redundancy, BLAST annotation; (4) insertion into the database.

#### Marker and sequence source

The current version of MoccaDB provides information regarding *Coffea *EST and genomic SSR markers retrieved from 11 published studies as well as original data (table 1). The database stores 638 markers, defined on 259 ESTs and 379 genomic sequences.

**Table 1:** Microsatellite markers, Sequence sources and original data

**Marker acronym used by the authors**	**Sequence type**	**Sequence origin (*Coffea*)**	**No of markers**	**Reference**		**Taxa tested**
CofEST-SSR	EST	*C. canephora × C. congensis*	9	(Bhat et al., 2005)	[24]	11 *Coffea *sp. 4 *Psilanthus *sp.
ES	EST	*C. canephora*	99	(Poncet et al., 2006)	[2]	7 *Coffea *sp.
				***Present study***		21 Rubiaceae sp.
SSR	EST	*C. canephora*	10	(Geromel et al., 2006)	[9]	*C. arabica*
DCM/CofEST-SSR	EST	*C. sp*.	9	(Aggarwal et al., 2007)	[7]	11 *Coffea *sp. 4 *Psilanthus *sp.
Ssr	EST	*C. canephora*	132	(Crouzillat et al., unpublished data)		
				***Present study***		3 *Coffea *sp.
M	Genomic	*C. arabica*	10	(Combes et al., 2000)	[25]	
M	Genomic	*C. arabica*	17	(Coulibaly et al., 2003)	[11]	2 *Coffea *sp.
CM	Genomic	*C. arabica*	9	(Baruah et al., 2003)	[26]	11 *Coffea *sp. 4 *Psilanthus *sp.
CFGA	Genomic	*C. arabica*	34	(Moncada et al., 2004)	[27]	*C. arabica*
M	Genomic	*C. arabica*	77	(Poncet et al., 2004)	[13]	6 *Coffea *sp.
DL	Genomic	*C. canephora*	8	(Leroy et al., 2005)	[19]	*C. canephora*
M	Genomic	*C. canephora*	213	(Poncet et al., 2007)	[1]	3 *Coffea *sp.
	Genomic	*C. arabica*	9	(Lashermes et al., unpublished data)		

**Total**			**638**			

Complete information on the origin of the data was reported such as laboratory, DNA library description, and, finally, reference of the published work. Polymerase chain reaction (PCR) primers, amplification conditions, and expected product sizes were directly retrieved from the publications, when available.

For most of the markers, nucleotide sequences were downloaded from GenBank databases  and stored in the database.

#### A unique set of markers

Most of the retrieved markers had been declared by their authors as designed on unigenes or, at least, on non-redundant DNA sequences. Nevertheless, to identify any redundancy due to the multiple origin of the data, all DNA sequences were checked for homology using the DNASTAR software package (Lasergene, Madison, WI, USA). The markers designed on sequences having a similarity percentage >90% were defined as "similar markers" in the database.

#### Annotation

Markers stored in the database are provided with a general SSR description: repeat motif and number, corresponding amino acid repeat if any, and, if known, SSR position on the sequence (coding region or UTR, as described in [2]).

Markers associated with experimentally described metabolic pathways (e.g. sucrose metabolism during coffee fruit development [9]) were integrated. Putative functions were predicted for all DNA sequences through similarity searches using BLASTx against GenBank protein databases [10].

#### Maps, transferability and diversity

Marker mapping data were retrieved from a published inter-specific *Coffea *linkage map [11] and can be visualized with CMap [12], integrated in MoccaDB.

The high transferability of SSR markers at evolutionarily conserved (orthologous) loci within the *Coffea *genus has been previously reported by different authors. For example, the percentage of transferability of SSR markers developed on *C. arabica *genomic DNA ranged from 72.7% for *C. liberica *Hiern to 86.4% for *C. pseudozanguebariae *Bridson [13].

Our previously published [2] and newly designed EST-SSR markers (Table 1), at a total of 99, were tested for amplification on a panel consisting of up to 21 Rubiaceae species belonging to the Cinchonoideae and Rubioideae subfamilies [14](Table 1). A new set of EST-SSR markers, provided by Crouzillat et al. (Table 1), was also tested on the following *Coffea *species: *C. canephora*, *C. heterocalyx *Stoff., and *C. pseudozanguebariae*. Only those showing a good and specific PCR amplification with an easy scoring of allele sizes were retained.

Both for markers retrieved from publications and for those designed and/or tested in this study, a maximum of available transferability-associated information was stored in the database: transferability status, amplification quality, information on the polymorphism (number and sizes of alleles within a given species, polymorphism information content (PIC) value).

### Database and Web application

**Figure 1:**
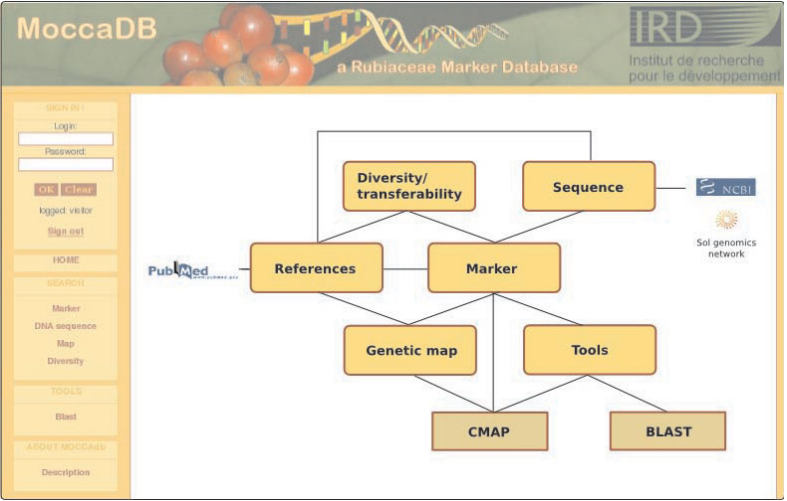
**Overview of the MoccaDB application**. MoccaDB integrates different data types, which are interconnected and linked to external resources and bioinformatic tools (CMap and BLAST).

MoccaDB has been designed for simple and efficient information search and retrieval. It is currently housed on a Linux Red Hat Enterprise server but is generally platform-independent. The database design has been carried out using the Unified Modeling Language (UML). MoccaDB is composed of two major components: a relational database created using open-access MySQL 5.0 and a PHP web application that communicates with the database. The web interface runs on the Apache 2 Web server. The PHP scripts dynamically execute complex SQL queries to retrieve data from the database according to user criteria and display them as a standard HTML output using CSS style sheets. MoccaDB also integrates bioinformatics tools such as BLAST [10] and CMap [12]. For an overview of the MoccaDB structure and interaction with the bioinformatics tools and external data sources, see Fig. 1.

The database contains mainly public but also some private data. The public data are accessible to any person connected to the MoccaDB Web site. To access to the private data of some scientific projects as well as to insert one's own data (markers, DNA sequences or mapping data) in the database, the user should open an account that is created with the permission of the scientific project manager. Several supplementary Web interfaces have thus been developed allowing the user an administrative access and database feeding.

## Utility

A user-friendly web interface has been developed to facilitate data retrieval according to specific user needs. One can search for markers, DNA sequences, maps and diversity data by using the corresponding multi-option query forms. The data can be viewed with a different degree of details, either as an overview (a list of search results), or as a detailed result page for a selected marker, sequence or map, with information on marker transferability, diversity and mapping. The experimental conditions, sequences and other relevant data are easily downloadable in different formats. Some additional information, like the construction of DNA libraries or description of the marker types, can be visualized with the help of pop up windows. Extensive, mostly bi-directional, hyperlinks are provided between the different data pages, thus facilitating the navigation within the web site (Fig. 1).

### Synthetic and downloadable information on annotated markers

Through the marker search page, markers can be directly searched by their names but the query can also be filtered by marker type, species and sequence origin, as well as by the availability of experimental data on their transferability and mapping.

The search results are displayed in the form of a table providing general information on each marker. The users can select any number of markers from this table and download them as an Excel file, together with additional optional information such as PCR experimental conditions, original DNA sequence, diversity/transferability or mapping data, depending on their scientific interests and future data utilisation. They can also access the detailed individual marker pages *via *the hyperlinks associated with each marker.

**Figure 2:**
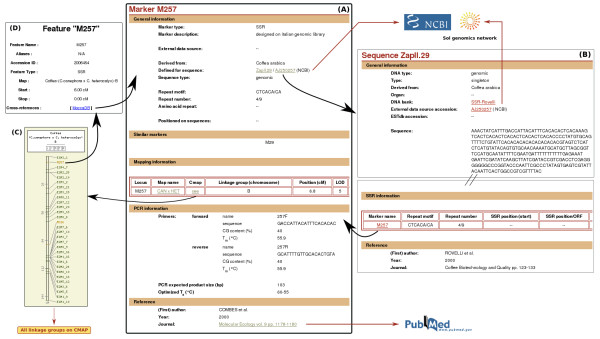
**Screenshots of a MoccaDB marker data pages**. (A) Marker detail page resulting from searching for the M257 marker. The result page provides general information about the SSR marker (e.g. repeat motif, repeat number) as well as the corresponding genomic sequence with database cross-references to NCBI and SOL (if available) and a hyperlink to the MoccaDB Sequence detail Page (B). The mapping information section provides information about marker locations on the genetic map, which links to the CMap viewer (C) and the CMap feature detail popup (D). The PCR information section may also provide details on marker assay conditions (forward and reverse primers, melting temperatures, predicted size of PCR product). The reference section gives publications related to this marker with links to Pubmed.

A typical individual marker page (Fig. 2) displays detailed information on diverse marker aspects: original sequence information, map location, transferability and/or intra- and inter-diversity, existence of "similar" markers developed on the same locus by other researchers, etc....

The genetically mapped markers can also be searched through the map search page. For each map, linkage groups can be displayed separately or together thanks to the CMap tool. A link associated with each SSR marker on the map brings the user back to the marker data page (Fig. 2).

### Functional markers directly targeting the expressed genes

**Figure 3:**
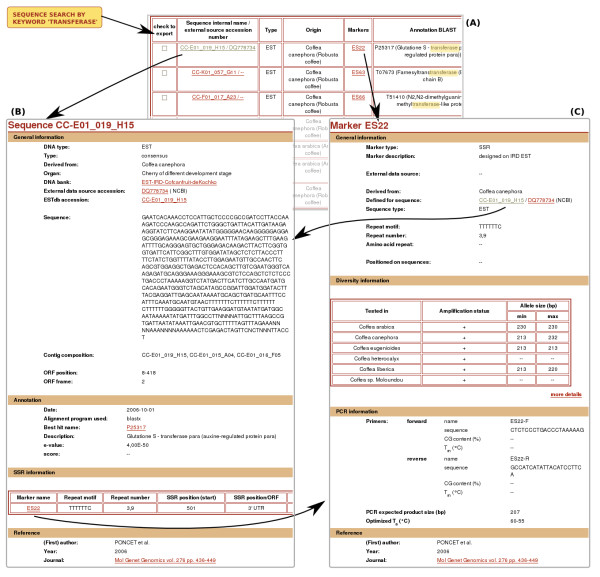
**Screenshots of sequence/putative function MoccaDB result pages**. Sequences can be searched by name, putative annotation. The search can be restricted using different criteria such as sequence origin or marker type. The group of screenshots shows an example sequence search using the keyword 'transferase' to find out what sequences have been "putatively" annotated with this term. (A) The result page displays sequences and related data resulting from searching for the annotation term 'transferase'. The tabular text summary lists all the sequences found, each line in the table presenting the sequence name and related information (sequence type and origin, marker name and BLAST annotation). The marker name and sequence name are respectively linked to the marker detail page (C) and to the sequence detail page (B). The user can select sequences and export them in FASTA format. The sequence detail page (B) displays all the associated information for that sequence which includes general information (e.g.: sequence type, DNA bank), annotation information, marker information and publications related to that sequence. Hyperlinks give access to associated data within MOCCAdb such as markers, DNA bank or link to external resources such as SOL or NCBI.

A user can search for sequences used to design the markers through the sequence search page. The query will optionally take into account the sequence name, species origin, sequence or marker type, and, more specifically, its putative function, namely a keyword in the BLAST annotation (e.g. transferase, Fig. 3). The sequence search page is especially useful when searching for "functional" markers linked to a particular metabolic pathway. Among different functions, the database is hosting markers associated with the sucrose metabolism [9].

The resulting searched sequences are displayed in a summary table with hyperlinks that give further access to sequence or marker data pages (Fig. 3). From this table, sequences selected by the user can be downloaded in a multi-fasta file to facilitate subsequent external analyses (BLAST search, clustering, etc...).

These functional markers could also be used in such studies as functional mapping, population analyses or association mapping.

### Transferable markers and polymorphism status

Transfer of genomic tools across species boundaries is crucial to assess variation in relevant germplasm and constitutes a unique tool to study orphan related species.

**Figure 4:**
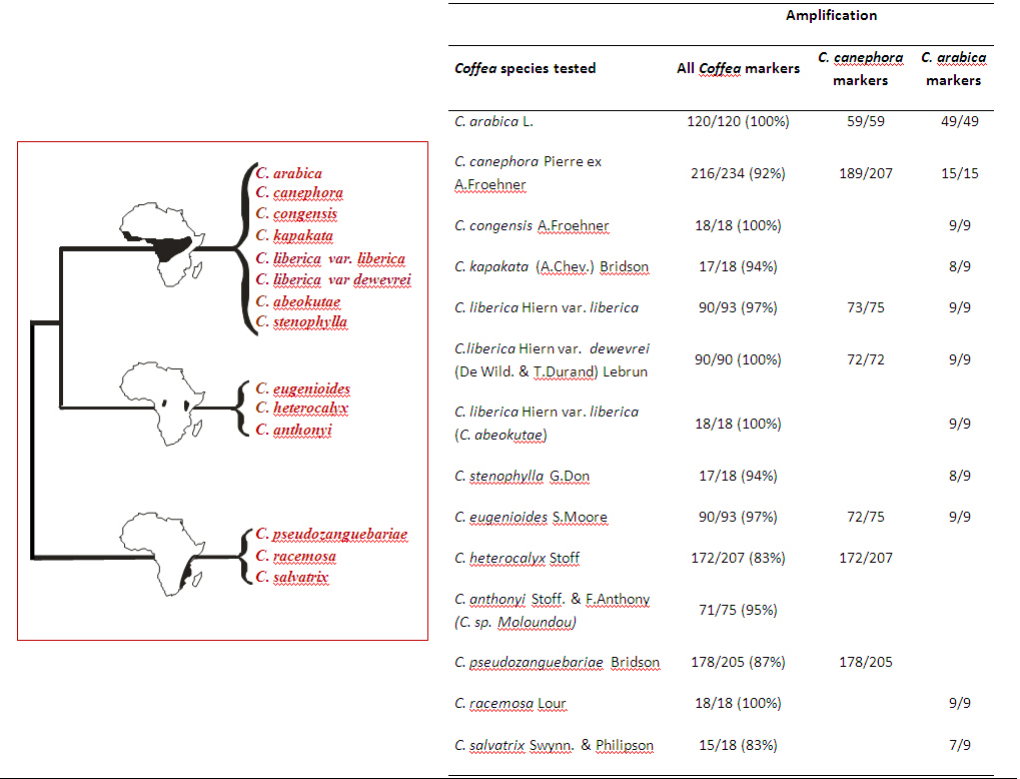
**Schematic phylogenetic tree adapted from **[21]**and number of successfully amplified/tested markers (percentage) observed for each species**. The information was extracted from MoccaDB database. Names of *Coffea *species follow [22,23].

In its current release, MoccaDB already gives access to valuable transferability data. In particular, of the *C. canephora *and *C. arabica *markers screened for cross-amplification and polymorphism, a minimum of 83% amplified alleles from any wild *Coffea *species, independently of its genetic relationship to both cultivated species (Fig. 4). Across the Rubiaceae family, many coffee markers were transferable to wild relatives of the Cinchonoideae subfamily, but only a fraction, maximum 12%, was transferable to distantly related genera in the Rubioideae subfamily (Table 2).

**Table 2:** Transferability to Rubiaceae species: efficiency of cross amplification of *Coffea *markers in other Rubiaceae genus (Nb species tested when over 1).

**Genus**	**% amplification**	**Nb of tested markers**	**Genus**	**% amplification**	**Nb of tested markers**
	
**Cinchonoideae**			*Paracephaelis*	32%	25 C
*Coffea *(14 sp.)	94%	Up to 207 C, up to 49 A	*Genipa*	24%	25 C
*Psilanthus *(4 sp.)	82%	9 A	*Chiococca*	16%	25 C
*Tricalysia *(2 sp.)	68%	25 C	*Ixora*	16%	25 C
*Bertiera*	56%	25 C	*Uncaria*	4%	25 C
*Pavetta*	48%	25 C	**Rubioideae**		
*Coptosperma*	40%	25 C	*Oldenlandia *(2 sp.)	12%	25 C
*Leptactina*	36%	25 C	*Psychotria *(4 sp.)	10%	25 C
*Tarenna*	36%	25 C	*Spermacoce *(2 sp.)	6%	25 C

Markers were developed on *C. canephora *(C) or *C. arabica *(A) sequences.

**Figure 5:**
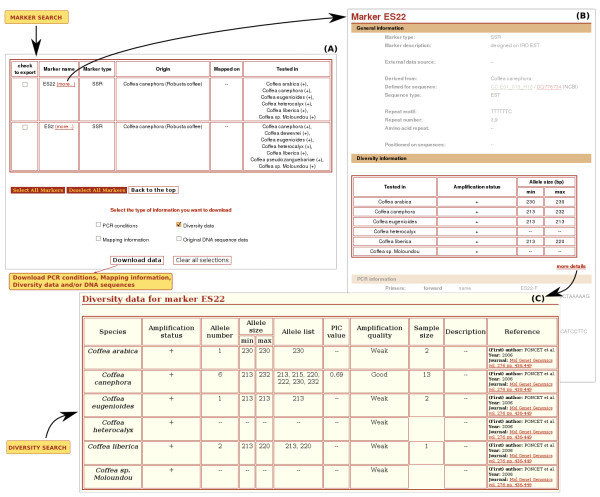
**Screenshots of transferability/diversity MoccaDB result pages**. (A) Marker search gives access to a synthetic results table with basic transferability data. Users can select markers and export related data on the amplified species and the corresponding allele size ranges (in bp) as Excel files. Hyperlinks on this result page gives access to the marker detail page (B), where this information can be directly visualized for each specific marker. Using the hyperlinks "more details", users can access additional details (C) on the transferability efficiency (presence or not of amplification products, quality of the amplification) and on the polymorphism status (number and sizes of the alleles, PIC values for each study). These diversity data can also be retrieved through the diversity search page and query filtered according to the species studied.

When working on one or more given species, the biologist can thus use the diversity query page to search markers that amplify these species, and eventually reveal inter-specific polymorphism (such as species-specific alleles) or intra-specific polymorphism (through the PIC parameter). Results for the searched markers are displayed in the form of a summary table (Fig. 5) with details on the marker transferability: species tested, amplification status, polymorphism, amplified allele range. These data will be particularly useful for researchers looking for an optimal polymorphic marker set for genotyping populations of a given species.

If the objective is the selection of markers for refining mapping in an inter-specific cross, or for discriminating two or more species, the user can identify diagnostic markers (i.e. with species-specific allele range) with known genetic map location or not.

A synthetic results table of these data can be obtained and downloaded from the marker search query page (Fig. 5).

### Bioinformatics tools and external links

CMap and the NCBI BLAST2.0 [10] were integrated into MoccaDB. Any given sequence can be searched for similarities against the MoccaDB sequences or updated public GenBank *Coffea *databases: (1) all *C. arabica *and/or *C. canephora *sequences; (2) *C. arabica *and/or *C. canephora *EST sequences; (3) *C. arabica *and/or *C. canephora *Genome Survey Sequences (GSS) sequences; (4) *C. arabica *and/or *C. canephora *«CoreNucleotide» (EST and GSS sequences not included).

External links connect MoccaDB to the NCBI genbank and Pubmed data, and to the SOL Genomics Network database [15] for some of the sequences developed on *C. canephora *by Crouzillat et al. (see table 1).

## Conclusion and perspectives

Contrary to some currently existing plant marker databases that contain predicted molecular markers (e.g. [16]), MoccaDB only stores validated markers provided with experimental protocols and related data. Indeed, we intended to centralize information on markers associated with single-copy loci, which can be reproducibly used for genetic analysis within the *Coffea *genus and related species.

Some *Coffea *genetic markers were made available by very few open and freely accessible database resources (Trieste [17], CIRAD [18]), but these resources are mostly limited to SSR data generated by their own hosting institute.

MoccaDB includes most of the publicly available data in addition to original data. As compared to the previously released databases, MoccaDB provides greater integrated information and specific features:

(1) Multiple options for data search and retrieval;

(2) Complete description of the markers, going from *in vitro *PCR amplification conditions, SSR and functional annotation of original DNA sequences and marker location on genetic maps, to cross amplification and diversity data;

(3) Synthetic and downloadable cross-amplification and diversity spreadsheet results to help the user in designing an optimal set of orthologous markers for genotypying or mapping studies in selected species and populations;

(4) Data selected by the user can be easily downloaded and used in laboratory experiments (PCR conditions, expected sizes, etc...) or for further analysis such as BLAST similarity searches of SSR-associated sequences (sequences provided in fasta format, etc...);

(5) Access is provided to integrated bioinformatics tools (CMap, BLAST), as well as to external hyperlinks to various public data sources (NCBI GenBank and Pubmed, SOL Genomics Network [15])

### MoccaDB evolution

In MoccaDB, a large amount of information is centralized and freely accessible to all users. A login system exists only for private project access and for data submission. To facilitate data integration, comma-separated values (csv) submission forms have been defined to allow automatical submission of data. More markers will be included in the database as and when they are made publicly available.

The database currently houses SSR markers from both genic and non-genic regions of the genome. Markers whose polymorphism is due to single-nucleotide polymorphism (SNPs), insertion/deletion (indels) or transposable elements are in the process of being developed and will be stored in MoccaDB in a near future.

Coffee has increasingly rich genetic and genomic resources including expressed sequences tags (ESTs) [e.g. [2]] and bacterial artificial chromosome (BAC) libraries [6,19]. Whole genome sequencing, genetic, physical and comparative maps are being developed. MoccaDB will be extended to include new data types, but also links to cytological maps and morphological data.

Systematic efforts have been initiated to generate PCR-based comparative genetic maps in several clades of plants, particularly in Solanaceae using Conserved Ortholog Set (COS) markers [20]. Data obtained in this family could be of benefit for wide comparative genomics studies including those of Rubiaceae species.

## Availability and requirements

The database is open and freely available

Project name: MoccaDB

Project home page: 

Operating system: Linux but functions also on Windows

Programming language: PHP5 (PHP4 compatible), (X)HTML, CSS2, JavaScript, AJAX, MySQL 5.0.45, SQL92

Other requirements: none

License: None required

## Competing interests

The authors declare that they have no competing interests.

## Authors' contributions

OP designed the project, designed and implemented the database, developed the web interfaces, FB designed the web interface. MC, AT and VV helped in analyzing the published markers, carried out the PCR amplification experiments and the genotyping. PDB identified/supplied plant material of Rubiaceae species from the greenhouses of the National Botanic Garden of Belgium. PDB and PH helped with the cross-amplification experiments and diversity analyses. CC helped in designing the database. AdK secured partial funding from the IRD-SPIRALES Board. AdK and SH coordinated the project. CT managed the project development, assisted in the designing of the database, performed database system administration, integrated the bioinformatics tools in the application. VP served as the principal investigator of the project, performed the data analysis, assisted in the designing of the database, and drafted the manuscript. All authors have contributed in the writing of the manuscript and have read and approved the final submitted version.
